# Insights into volatile flavoromics and microbiomics for sensory quality improvement of flue-cured tobacco leaves fermented with *Bacillus subtilis* DB-15

**DOI:** 10.1186/s40643-026-01124-2

**Published:** 2026-08-31

**Authors:** Yanxu Guo, Junxi Wang, Guiyuan Huang, Ningning Hou, Haiyang Pan, Wei Ding

**Affiliations:** Technical Research Center, China Tobacco Sichuan Industrial Co., Ltd., 56 Chenglong Road, Chengdu, 610000 China

**Keywords:** *Bacillus subtilis* DB-15, Tobacco leaves fermentation, Volatile flavor compounds, Microbial community, Correlation network

## Abstract

Low-quality tobacco leaves generally exhibit undesirable sensory characteristics due to excessive macromolecule accumulation and insufficient aroma release. Fermentation with functional microorganisms to improve tobacco quality is a green and efficient strategy. This study employed volatile flavoromics and microbiomics to investigate the flavor basis and underlying microbial regulatory mechanism for quality improvement by *Bacillus subtilis* DB-15. Results showed that *B. subtilis* DB-15 fermentation significantly degraded starch, protein, and cellulose, and comprehensively enhanced sensory quality by increasing aroma and sweetness while reducing irritation and off-flavors. Subsequently, a total of 94 volatile compounds were identified by HS-SPME-GC-MS. *B. subtilis* DB-15 promoted the enrichment of esters and heterocycles, and inhibited harmful substances such as short-chain fatty acids and nicotine. Microbiomics analysis indicated that inoculated *B. subtilis* DB-15 dominated the bacterial community and reshaped its structure, whereas fungal community succession was mainly driven by water addition rather than *Bacillus* inoculation. Functional prediction suggested a higher relative abundance of bacterial pathways related to carbohydrate and amino acid metabolism in the *Bacillus* inoculation group, whereas pathways related to terpenoid and polyketide metabolism showed lower predicted abundances. Correlation network analysis showed that *Bacillus* was positively correlated with sweet and fruity aroma compounds but negatively correlated with irritant acids, suggesting a potential contribution to improved sensory coordination. This study reveals the mechanism of sensory improvement by *B. subtilis* DB-15 fermentation from dual-omics perspective, providing a reliable technical strategy for upgrading low-quality tobacco resources.

**Graphical abstract:**

**Figure:**
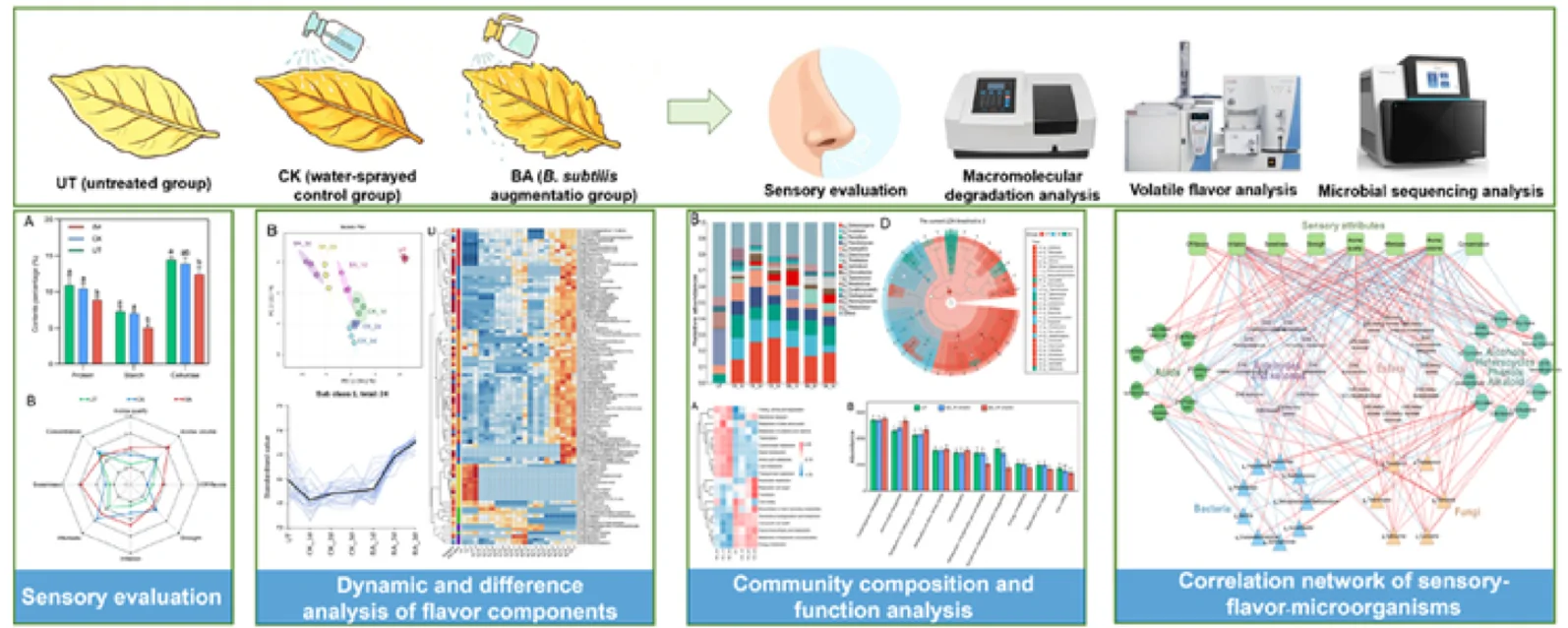

**Supplementary Information:**

The online version contains supplementary material available at https://doi.org/10.1186/s40643-026-01124-2.

## Introduction

Tobacco is an important non-food cash crop globally and cultivated in more than 100 countries (Abdellah et al. 2025; Wei et al. 2025). As the core raw material of the tobacco industry, tobacco quality directly determines the grade and sensory performance of cigarette products. After harvesting, tobacco leaves generally undergo a series of processing procedures including curing, conditioning, aging, and fermentation to achieve coordinated transformation of chemical components and improvement of sensory quality (Wu et al. 2022; Yang et al. 2023). In practical production, due to the influences of planting environment, harvesting time, curing technology and aging conditions, a large number of low-quality tobacco leaves with inherent quality defects are annually generated and difficult to utilize (Abdellah et al. 2025). They generally exhibit insufficient aroma quantity, poor aroma quality, strong irritation, and obvious offensive and undesirable odors, which greatly limit their industrial availability and lead to the waste of agricultural resources (Ahmed et al. 2024).

Low-quality tobacco leaves with inadequate aging or incomplete fermentation are mainly characterized by the insufficient degradation and transformation of macromolecular substances such as starch, protein and cellulose (Ma et al. 2024; Zhang et al. 2023). These substances tend to produce burnt, bitter, astringent, and irritating gases during combustion, accompanied by distinct undesirable odors, irritating smoke, and unclean aftertaste, as well as weak aroma, which severely reduces the smoking experience (Gong et al. 2023; Ning et al. 2023). At present, strategies including physical modification (Xu et al. 2025), chemical regulation (Li et al. 2024a), enzymatic treatment (Shu et al. 2026; Xu et al. 2024), and functional microbial fermentation have been widely applied to improve tobacco leaf quality (Zhang et al. 2025b). Nevertheless, physical modification presents limited improvement effects and a long processing cycle, while chemical regulation easily causes substance residues and safety concerns, and enzymatic treatment suffers from high application cost and a single regulatory effect, which makes them difficult to meet the industrial requirements for green quality improvement. In contrast, functional microbial fermentation has become a promising strategy for upgrading low-quality tobacco leaves owing to its prominent advantages including environmental safety, high efficiency, mild action, low cost, and the ability to directionally improve flavor coordination (Song et al.^2026^; Zhang et al. 2025b).

In recent years, extensive studies have been conducted to explore the microbial community structure and metabolic functions during tobacco fermentation (Li et al. 2020; Tao et al. 2024). It has been confirmed that diverse microorganisms, including bacteria such as *Bacillus*, *Pseudomonas*, and *Sphingomonas Staphylococcus*, *Kocuria*, fungi such as *Wallemiales*,* Aspergillus*, *Penicillium*, *Cladosporium* and *Candida* act as the core groups in the natural fermentation system (Zhang et al. 2025b; Zheng et al. 2026). These microbial communities jointly participate in the degradation of macromolecules such as proteins, pectin, and cellulose (Tao et al. 2024), as well as the transformation of flavor compounds including *Maillard* reaction products and terpenoids (Wei et al. 2024). On this basis, functional microorganisms screened from natural fermentation systems, for example *Bacillus* (Liu et al. 2025), *Sphingomonas* (Wu et al. 2025) and *Aspergillus* (Zhao et al. 2024), can rapidly and effectively improve the aroma quality and sensory comfort of tobacco leaves. They achieve this by secreting extracellular enzymes such as proteases, amylases, and pectinases to degrade macromolecules, synthesizing aroma active compounds like higher alcohols, esters, and ketones, and meanwhile reducing harmful components such as nicotine and tobacco specific nitrosamines (Li et al. 2024b). Nevertheless, most current studies merely concentrate on the sensory performance of single-strain fermentation or the detection of volatile flavor compounds. Relatively few investigations have systematically explored the intrinsic correlations among the succession of microbial community structure and its metabolic functions, flavor substance metabolism, and sensory quality. The potential mechanism underlying the improvement of tobacco leaf quality defects by functional microorganisms remains insufficiently understood.

In our previous work, a functional strain *Bacillus subtilis* DB-15 with strong macromolecule-degrading ability was screened and identified through morphological observation and partial 16 S rRNA sequencing. This strain displayed remarkable aroma-improving effects on low-grade flue-cured tobacco leaves. Accordingly, this study used tobacco leaves that still exhibited quality defects after two years of natural aging as experimental materials. Three groups were set up: untreated group (UT), water-sprayed control group (CK), and *B. subtilis* DB-15 augmentation group (BA), and dynamic samples were collected during fermentation. Combined with volatile flavoromics and microbiomics, we analyzed the dynamic changes of flavor compounds, the succession characteristics of microbial communities, and differences in metabolic functions during fermentation. Furthermore, correlation analyses were conducted to explore the associations among sensory quality, key flavor compounds, and characteristic microorganisms. This study aims to clarify the flavor metabolite basis for the improvement of sensory quality by *B. subtilis* DB-15, provide insights into the potential microbial regulatory mechanism underlying quality enhancement, and offer theoretical references and practical technical support for the green and efficient resource utilization of low-quality tobacco leaves.

## Materials and methods

### Strain information and tobacco leaf materials

*Bacillus subtilis* DB-15 used in this study was isolated from naturally aged tobacco leaves, and has been long-term preserved in the strain library of Technical Research Center, China Tobacco Sichuan Industrial Co., Ltd. with preservation number BS-Tob15. The strain was identified based on its morphological, physiological, and biochemical characteristics, as well as partial 16 S rRNA gene sequencing using primers 27 F and 1492R. The obtained sequence was submitted to NCBI BLAST, which showed 98.29% identity to the 16 S rRNA gene of *Bacillus subtilis* strain HRY011 (GenBank accession No. PX623649.1), confirming its identification as *B. subtilis*. For fermentation inoculation, the strain was aerobically activated in LB medium at 37 °C with shaking at 220 r/min for 24 h. The cell concentration was adjusted to about 1 × 10⁷ cells/mL with sterile deionized water using a microbial cell analyzer (Countstar Mira FL Pro, ALIT Life Science, Shanghai, China) for subsequent inoculation. The experimental tobacco leaf materials were upper leaf samples with strong aroma type, which had been stored in the Duobao warehouse of China Tobacco Sichuan Industrial Co., Ltd. for two years of natural aging but still exhibited obvious quality defects. Prior to fermentation, all tobacco leaves were sealed in bags and stored at − 40 °C to preserve their initial state.

### Tobacco fermentation and sampling

Three groups were established: untreated group (UT), water-sprayed control group (CK), and *B. subtilis* DB-15 augmentation group (BA). The fermentation conditions of tobacco leaves were determined referring to previous studies with appropriate modifications (Wu et al. 2021). For each treatment, three independent biological replicates were prepared, with 200 g of tobacco leaves per replicate. Tobacco leaves were placed in sterilized wide-mouth glass jars. For the BA group, a bacterial suspension containing approximately 1 × 10⁷ cells/mL was uniformly sprayed onto tobacco leaves at a ratio of 10% (v/w), whereas an equal volume of sterile water was sprayed in the CK group. The opening of each jar was covered with four layers of sterilized gauze to allow passive air exchange while minimizing external contamination. All samples were incubated at 30 °C with 80% relative humidity for fermentation. Samples were collected from each biological replicate on days 1, 2, and 3 of fermentation. At each time point, the samples were divided into two portions. One portion (approximately 10 g per biological replicate) was directly sealed and stored at − 80 °C for subsequent microbiomic sequencing. The other portion was immediately heat-inactivated at 70 °C for 30 min to terminate fermentation, equilibrated at 60% relative humidity for 24 h, and stored at − 80 °C for volatile flavor compound analysis and sensory evaluation. The UT group (day 0) was subjected to the same heat-inactivation procedure immediately after moisture adjustment to represent the true starting point. For sensory evaluation, only the UT group (day 0) and the BA and CK groups at day 3 (the end of fermentation) were assessed.

### Determination of macromolecular components in tobacco leaves

Starch content was determined according to the tobacco industrial standard YC/T 216-2013 using the iodine colorimetric method. Briefly, tobacco samples were extracted with 80% ethanol-saturated sodium chloride solution to remove impurities, followed by extraction with 40% perchloric acid to solubilize starch. Under acidic conditions, starch was hydrolyzed to dextrins, which reacted with iodine to form a blue colored complex measured at 570 nm. Protein content was determined following the standard YC/T 249–2008 using the continuous flow method (Zhang et al. 2024), in which protein nitrogen was precipitated with acetic acid, digested, and reacted with reagent under alkaline conditions to form an indigo blue compound measured at 660 nm. Cellulose content was determined using the anthrone-sulfuric acid colorimetric method, in which cellulose is hydrolyzed to glucose by concentrated sulfuric acid and then dehydrated to hydroxymethylfurfural, which reacts with anthrone to produce a blue-green complex measured at 620 nm. Each sample was analyzed in three technical replicates. Significant differences were analyzed by one-way ANOVA followed by Tukey’s multiple comparison test (*p* < 0.05).

### Determination of macromolecular degradation enzyme activity of *B. subtilis* DB-15

The crude enzyme solution was obtained by centrifuging the *B. subtilis* DB-15 liquid culture at 4 °C and 7000 rpm (6190 × *g*) for 10 min. The enzymatic activities of α-amylase, neutral protease and cellulase were determined according to the previous methods (Shan et al. 2025). Briefly, amylase and cellulase activities were measured using the 3,5‑dinitrosalicylic acid (DNS) method, with 1% soluble starch and 1% sodium carboxymethyl cellulose as substrates, respectively. The reaction mixtures were incubated at 35 °C for amylase and at 50 °C for cellulase for 30 min, and the absorbance was measured at 540 nm. One unit (U) of amylase or cellulase activity was defined as the amount of enzyme required to produce 1 µg of glucose per milliliter of enzyme solution per minute under the assay conditions. Neutral protease activity was determined using the Folin‑phenol method with 1% casein as substrate. The reaction was carried out at 40 °C for 10 min, and the absorbance was measured at 680 nm. One unit (U) of protease activity was defined as the amount of enzyme required to hydrolyze casein and produce 1 µg of tyrosine per milliliter of enzyme solution per minute under the assay conditions. All assays were performed in triplicate.

### Sensory quality evaluation

Referring to the Chinese industrial standards YC/T 415–2011 Sensory evaluation method for tobacco in-process materials and YC/T 496-2014 Sensory comfort evaluation method for cigarettes, eight core indexes were selected to evaluate the sensory quality of tobacco leaves: aroma quality (the superiority and characteristic preference of aroma), aroma volume (the abundance and fullness of aroma), off-flavors (undesirable odors in smoke excluding inherent tobacco aroma; higher scores indicate fewer off-flavors), irritation (stimulus sensation of smoke on the oral cavity, nasal cavity and throat; higher scores represent weaker irritation), concentration (the thickness intensity of smoke perceived in the mouth), strength (the impact intensity of smoke on the throat), aftertaste (the residual taste and comfort in the oral cavity after smoking), and sweetness (the sweet and mellow texture presented in smoke and aftertaste).

The sensory evaluation panel consisted of 10 professional assessors with official qualification for tobacco sensory evaluation. All panelists are experienced tobacco sensory evaluators from China Tobacco Sichuan Industrial Co., Ltd., with many years of professional practice. All samples were assessed through randomized blind evaluation, and each index was scored continuously on a 0 to 9 scale (0 = extremely poor and completely unacceptable, 3 = relatively poor, 5 = moderate, 7 = good or comfortable, and 9 = excellent or perfect experience), with higher scores representing better sensory performance. Specifically, higher scores for irritation and off-flavors indicate weaker irritation and lighter miscellaneous odors, respectively. The average score of each index from all assessors was adopted to plot the sensory radar chart. The total score of all indexes was calculated as the comprehensive score to characterize the overall sensory quality of tobacco leaf samples. To assess inter-panelist reproducibility, the intraclass correlation coefficient (ICC, two-way random effects model for absolute agreement) was calculated for each sensory attribute. The specific scoring information for sensory evaluation is shown in Supplementary Table S1.

### Analysis of volatile flavor compounds

Volatile flavor compounds were analyzed using headspace solid-phase microextraction (HS-SPME) coupled with gas chromatography-mass spectrometry (GC-MS) according to previous report with minor modifications (Zhang et al. 2025a). In brief, 1.5 g of tobacco powder was placed into a 20 mL headspace vial for volatiles extraction using a 50/30 µm DVB/CAR/PDMS fiber (Sigma-Aldrich, USA) at 60 °C for 30 min. After extraction, the fiber was immediately desorbed in the GC-MS injector at 250 °C for 5 min. Chromatographic separation was performed on a TG-5 column (60 m × 0.25 mm × 0.25 μm; Thermo Scientific, Waltham, MA, USA). Helium was used as the carrier gas at a constant flow rate of 1 mL/min. The oven temperature program was as follows: held at 40 °C for 2 min, increased to 250 °C at 10 °C/min, and maintained at 250 °C for 5 min. The transfer line and ion source temperatures were set at 280 °C and 300 °C, respectively. Mass spectra were acquired in electron impact (EI) mode at 70 eV, scanning over a range of m/z 33–400. Volatile compounds were putatively annotated by matching their mass spectra against the NIST mass spectral library and by comparing their calculated retention indices (RIs) with available reference information. A C7-C30 n-alkane standard mixture was analyzed under the same GC-MS conditions, and the RI of each volatile compound was calculated based on the retention times of the adjacent n-alkanes (Figure S1). The calculated RIs are listed in Supplementary Table S2. Due to the unavailability of authentic standards for all detected volatile compounds, compound identities should be regarded as putative annotations. For quantification, 10 µL of ethyl decanoate (0.2215 mg/mL) was added as an internal standard prior to extraction. The information of 94 identified volatile flavor compounds is presented in Supplementary Table S2.

### Microbial DNA extraction and sequencing analysis

The DNA extraction and sequencing analysis of surface microorganisms from tobacco leaves are as follows: In brief, tobacco leaf samples (5 g) were ground into powder in a pre-chilled mortar with liquid nitrogen and then mixed with 100 mL of sterile physiological saline solution (0.9% NaCl) in a 250 mL conical flask. The mixture was agitated on a shaker at 10 °C and 220 rpm for 3 h. After filtering the tobacco debris through four layers of gauze, the microbial cells were pelleted by centrifugation at 4 °C and 7,000 rpm for 15 min. The bacterial precipitate was collected. It should be noted that the saline-washing and shaking procedure used to detach microorganisms from tobacco leaves may introduce recovery bias, as microbial taxa may differ in their attachment strength to the leaf surface. However, the identical protocol applied to all samples minimizes its impact on relative comparisons among groups.

Total microbial DNA was extracted using the DNeasy PowerSoil Pro Kit, and the purity and concentration of DNA were assessed through agarose gel electrophoresis. Each sample underwent three biological replicates, and the extracted DNA samples were stored at − 80 °C for subsequent amplicon sequencing analysis. The extracted DNA was used as a template. The bacterial 16 S rRNA region was amplified with primers 338 F and 806R, while the fungal ITS1 region was amplified with primers ITS1F and ITS2R. Purified amplicons were pooled in equimolar amounts and paired-end sequenced (2 × 250 bp) on an Illumina HiSeq 2500 platform (Illumina, San Diego, USA). Raw sequences were processed using QIIME2 software, which involved trimming low-quality regions, demultiplexing sample data based on barcodes, and removing barcodes and primer sequences to obtain preliminary quality-controlled data. The UCHIME algorithm was used to detect and remove chimeric sequences, resulting in valid data. Valid data were clustered into ASVs at 99% similarity. Species classification annotation was performed using the q2-feature-classifier plugin against the SILVA database for bacteria and the UNITE database for fungi. The raw sequencing reads have been deposited in the China National Center for Bioinformation (CNCB) database under BioProject ID PRJCA068606. Microbial metabolic potential was predicted using PICRUSt2. Bacterial functions were annotated based on the KEGG database. Fungal functions were annotated against the MetaCyc database due to its broader coverage of eukaryotic metabolic pathways. Level 2 functional pathways were used to compare differences in community metabolic potential among groups. The PICRUSt2 results were interpreted as predicted microbial functional potential based on 16 S rRNA gene and ITS amplicon data, rather than direct measurements of pathway expression or metabolic activity.

### Statistical analysis and visualization

Differences in macromolecular contents were analyzed by one-way ANOVA with Tukey’s test. Differences in microbial metabolic potential were analyzed by one-way ANOVA with Duncan’s multiple comparison test. Alpha diversity differences among groups were analyzed using the Kruskal-Wallis’s test. Principal component analysis (PCA), partial least squares discriminant analysis (PLS-DA), dynamic heatmap and K-means clustering were used to analyze flavor compounds. For heatmap visualization of volatile compounds, Z-score normalization was applied to each compound across all samples to facilitate the comparison of relative accumulation patterns. Principal coordinates analysis (PCoA) was used to reveal microbial β-diversity. LEfSe analysis was conducted among the UT, CK, and BA groups using the all-against-all multiclass comparison strategy. The Kruskal-Wallis test was used for initial screening, and taxa with an LDA score > 2.0 were retained. No subclass variable was included. A Spearman correlation heatmap was generated between sensory attributes and 40 key differential volatile compounds selected based on VIP > 1 and Kruskal-Wallis test *p* < 0.05 among the UT, CK_3d, and BA_3d groups. A bipartite network was constructed between these key volatile compounds and LEfSe-identified characteristic microorganisms (LDA score > 2), retaining correlations with |r| > 0.8 and Benjamini-Hochberg FDR *p* < 0.05.

## Results and discussion

### Macromolecule degradation and sensory quality evaluation of *B. subtilis* DB-15 fermented tobacco leaves

**Fig. 1:**
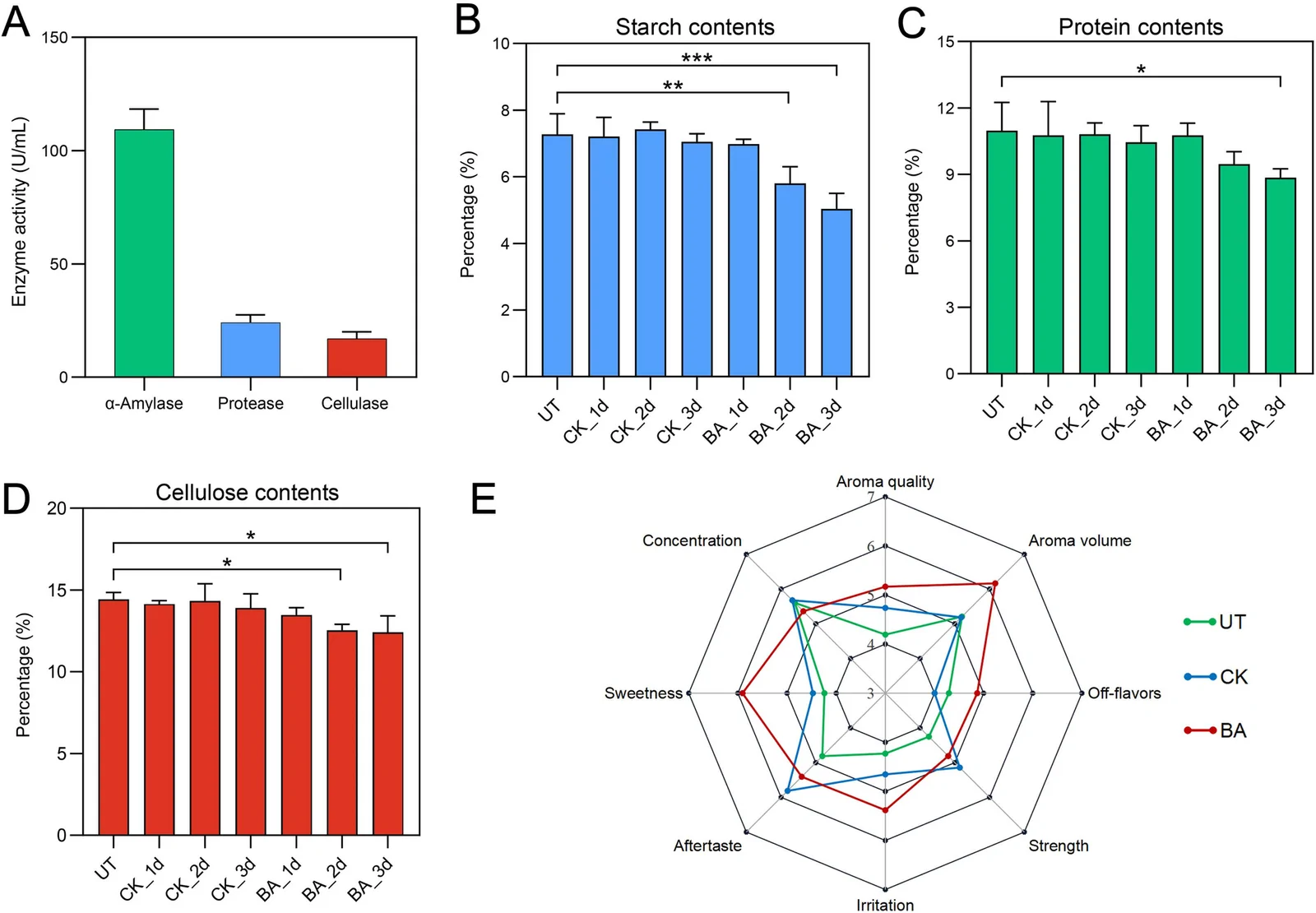
Enzyme activities of *B. subtilis* DB-15 liquid culture and dynamic changes of macromolecule contents and sensory quality of tobacco leaves in different treatment groups. **A** The α-Amylase, protease, and cellulase activities of the *B. subtilis* DB-15 liquid culture. **B**–**D** Dynamic changes of starch (**B**), protein (**C**), and cellulose (**D**) contents during fermentation in the untreated group (UT), water-sprayed control group (CK), and *B. subtilis* DB-15 augmentation group (BA) over 0, 1, 2, and 3 days. **E** Sensory evaluation radar chart of tobacco leaves in UT, CK, and BA groups at the end of fermentation. Data are presented as mean scores from 10 panelists. Statistical comparisons (mean ± SD, pairwise t-test) and inter-panelist reproducibility (ICC) are provided in Supplementary Table S1. *Note* *p < 0.05, **p < 0.01, ***p < 0.001 compared with the UT group at the same time point (one-way ANOVA followed by Tukey’s test)

Low-quality aged tobacco leaves are generally characterized by excessive accumulation of macromolecules such as starch and protein (Wu et al. 2021). Excessively high levels of starch and protein may not only lead to insufficient combustion, offensive taste, and pungent irritation in the smoke (Gong et al. 2023), but also weaken the sweetness and aroma expression of tobacco. To investigate the large-molecule degradation ability of *Bacillus subtilis* DB-15 and its effect on improving the sensory quality of tobacco, the extracellular enzyme activities in its liquid culture were first determined. As shown in Fig. 1A, the strain exhibited clear α-amylase (109.4 ± 7.35 U/mL), protease (24.2 ± 2.71 U/mL), and cellulase (17.0 ± 2.50 U/mL) activities, confirming its intrinsic potential to hydrolyze starch, protein, and cellulose. To further evaluate the dynamic degradation patterns during tobacco fermentation, we monitored the contents of starch, protein, and cellulose over the entire fermentation time course (days 0, 1, 2, and 3) in the untreated group (UT), water‑sprayed control group (CK), and *B. subtilis* DB‑15 augmentation group (BA) (Fig. 1B-D). Compared with the UT group, the contents of protein, starch, and cellulose in the CK group showed slight but non-significant decreases (Fig. 1B-D). By contrast, the BA group exhibited continuous and time-dependent degradation of three macromolecules from day 1 to day 3. Specifically, starch content decreased from 7.27 to 5.02%, protein content decreased from 10.98% to 8.86%, and cellulose content decreased from 14.43 to 12.42% by the end of fermentation, all of which were significantly lower than those in the UT and CK groups (*p* < 0.05) (Fig. 1B–D). These results demonstrated that inoculation with *Bacillus* DB-15 can efficiently degrade macromolecular components including protein, starch, and cellulose in tobacco leaves in a time-dependent manner, consistent with the enzyme activities detected in the liquid culture and previous reports that *Bacillus* can efficiently degrade macromolecular components by secreting extracellular hydrolases (Wu et al. 2021).

Sensory evaluation results revealed that the CK treatment partially improved certain smoke indicators, whereas the BA treatment displayed clear advantages across multiple core sensory dimensions (Fig. 1E & Supplementary Table S1). Specifically, compared with the UT group, strength (5.14 points in CK) and aftertaste (5.82 points in CK) increased significantly by 21.0% and 20.8% (*p* < 0.05), respectively, while the improvement in other attributes was limited. Notably, the off-flavors score (4.00 points in CK) declined relative to the UT group (4.29 points in UT), suggesting that water-only fermentation may aggravate the release of undesirable flavors due to uncontrollable factors (Fig. 1E & Supplementary Table S1). In comparison, the BA treatment displayed clear advantages across multiple core sensory dimensions. The scores of aroma quality (5.17 points in BA), aroma volume (6.16 points in BA), irritation (5.38 points in BA; higher score indicates weaker irritation), and sweetness (5.90 points in BA) were significantly improved by 23.4%, 18.2%, 27.4%, and 39.4%, respectively, relative to the UT group (*p* < 0.05) (Fig. 1E & Supplementary Table S1). Meanwhile, the off-flavors score (4.87 points in BA; higher score indicates fewer off-flavors) increased by 20.8% compared with the CK group, effectively remedying the defects of the CK treatment. Of note, although the concentration (5.36 points in BA) and strength (4.81 points in BA) of the BA group were slightly lower than those of the CK group, the overall coordination was significantly improved (Fig. 1E). Inter-panelist reproducibility, assessed by the intraclass correlation coefficient (ICC), ranged from 0.628 to 0.881 for most attributes (excluding “Concentration”), indicating “Good” to “Moderate” consistency among the 10 panelists (Supplementary Table S1). The relatively lower ICC for “Concentration” (0.249) was likely due to the close proximity of scores among the three sample groups, which reduced between-sample variance, rather than indicating poor panelist reliability. These results indicate that *B. subtilis* DB-15 may comprehensively enhance the sensory quality of tobacco leaves by strengthening macromolecule degradation, reducing the precursors of undesirable flavors at the source, releasing abundant flavor precursors to improve aroma quality, aroma volume, and sweetness, and diminishing the sources of off-flavors.

### Influence of *B. subtilis* DB-15 fermentation on the volatile flavor compounds of tobacco leaves

#### Dynamic changes of volatile flavor compounds

**Fig. 2:**
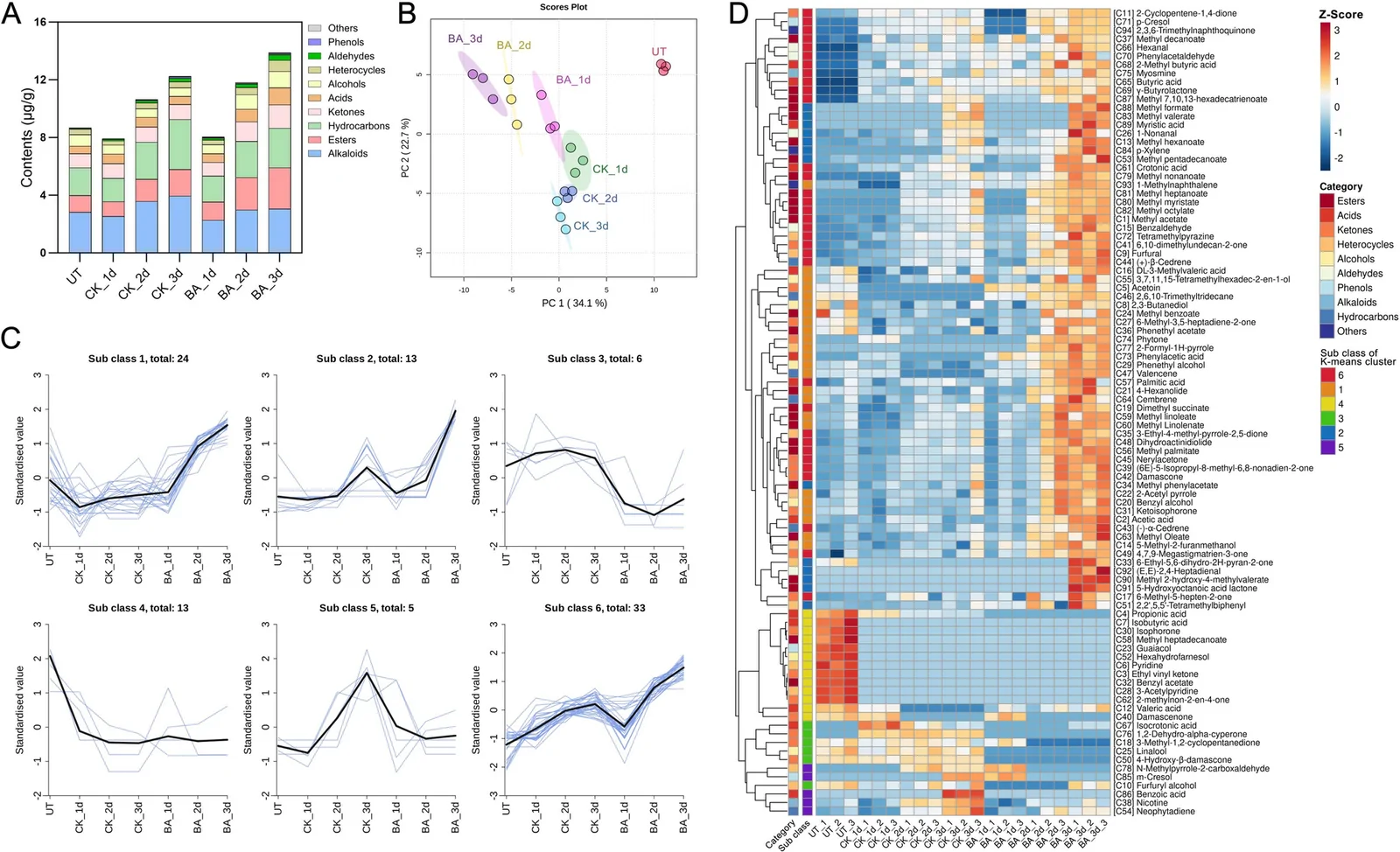
Dynamic changes of volatile flavor compounds during fermentation. **A** Stacked bar chart showing the contents of volatile flavor compounds in UT, CK and BA groups during fermentation. **B** PCA score plot of volatile profiles. **C** K-means clustering analysis of the dynamic patterns of 94 volatile compounds **D** Heatmap of 94 volatile compound dynamics

On the basis of clarifying the characteristics of macromolecule degradation and sensory quality improvement, volatile flavor metabolomics was further applied to explore the material basis underlying the enhancement of flavor quality by BA fermentation. A total of 94 volatile flavor compounds were identified in tobacco leaves by HS-SPME-GC-MS, including 26 esters, 18 ketones, 13 acids, 12 heterocycles, 6 alcohols, 6 hydrocarbons, 5 aldehydes, 3 phenols, 2 alkaloids, and 3 other compounds (Supplementary Tables 2 & Fig. 2).

In terms of compound concentrations (Fig. 2A), the total contents of volatile flavor compounds in both the CK and BA groups gradually increased during fermentation and were distinctly higher than those in the untreated group (UT). Specifically, the concentrations of alkaloids and hydrocarbons in the CK group continuously increased with fermentation time, reaching 3.95 µg/g and 3.46 µg/g at 3 days, representing increases of 39.2% and 79.7% compared with the UT group. In the BA group, the concentrations of esters, ketones, alcohols, and heterocycles showed steady upward trends during fermentation, which were higher than those in the UT group and the contemporaneous CK group (Fig. 2A). Notably, the concentration of esters exhibited the most pronounced increase, reaching 2.86 µg/g at 3 days, which was 2.48‑fold and 1.55‑fold that of the UT group (1.15 µg/g) and CK group (1.86 µg/g), respectively. These results indicate that inoculation with *B. subtilis* DB-15 may effectively promote the synthesis and transformation of volatile flavor compounds in tobacco leaves, providing a direct material basis for improving sensory quality. Subsequently, PCA was performed to evaluate the overall differences in volatile flavor metabolic profiles among tobacco samples (Fig. 2B). The PCA score plot (PC1 and PC2 together explained 56.8% of the total variance) showed clear separation between untreated tobacco samples and fermented samples of the CK and BA groups. Moreover, samples from the CK and BA groups diverged noticeably with fermentation time and formed well defined dynamic clusters (Fig. 2B). These results demonstrate that both water only and BA inoculation fermentation reshaped the flavor metabolic profile of tobacco, but the two treatments followed strikingly different flavor evolution patterns.

Furthermore, K-means clustering and concentration heatmaps were used to visualize the distribution patterns of flavor compounds across different samples (Fig. 2C,D). K-means clustering divided the 94 volatile flavor compounds into six subclusters (Fig. 2C), and compounds in each subcluster displayed distinctly different accumulation patterns among treatments and fermentation stages. Compounds in sub class 1, 2, and 6 showed an overall upregulated and enriched trend during BA fermentation (Fig. 2C), representing the most prominent responsive flavor components induced by *B. subtilis* DB-15. These sub class were abundant in various aroma-active compounds, including esters (such as [C36] phenethyl acetate, [C59] methyl linoleate, [C60] methyl linolenate, and [C63] methyl oleate), heterocycles (such as [C14] 5-methyl-2-furanmethanol, [C22] 2-acetyl pyrrole, and [C77] 2-formyl-1 H-pyrrole), alcohols (such as [C8] 2,3-butanediol, [C20] benzyl alcohol, and [C29] phenethyl alcohol), as well as ketones (such as [C31] ketoisophorone and [C74] phytone) (Fig. 2C,D). Esters and alcohols generally possess fruity and floral aroma characteristics (Holt et al. 2019), while heterocyclics often contribute roasted and baked notes (Al-Khalili et al. 2025), which may contribute to the improved aroma quality, aroma volume, and sweetness of tobacco in the BA group.

In contrast, sub class 4 and 5 (18 compounds in total) represented the downregulated compounds during BA fermentation (Fig. 2C,D). Sub class 4 (13 compounds) was dominated by short-chain fatty acids (e.g., [C4] propionic acid, [C7] isobutyric acid, [C12] valeric acid), several ketones (e.g., [C3] ethyl vinyl ketone, [C30] isophorone, [C40] damascenone), and phenols (e.g., [C23] guaiacol). These compounds were abundant in the UT group and showed downward trends with both BA and CK fermentation. The five compounds in sub class 5 peaked at CK_3d, including [C38] nicotine, [C85] m-cresol, and [C86] benzoic acid. Nicotine is generally related to smoke strength (McKinney et al. 2014), and its increased content in the CK group may explain the higher strength observed in this group. Compounds in sub class 3 (6 compounds) showed an overall first-increasing then decreasing trend. Several heterocyclic and alcoholic compounds (e.g., [C10] furfuryl alcohol, [C18] 3-methyl-1,2-cyclopentanedione, [C25] linalool) increased slightly in the CK group during fermentation but decreased sharply on day 1 of BA fermentation, rose slightly on day 3, and finally remained at a relatively low level.

#### Identification of key differential flavor compounds

**Fig. 3:**
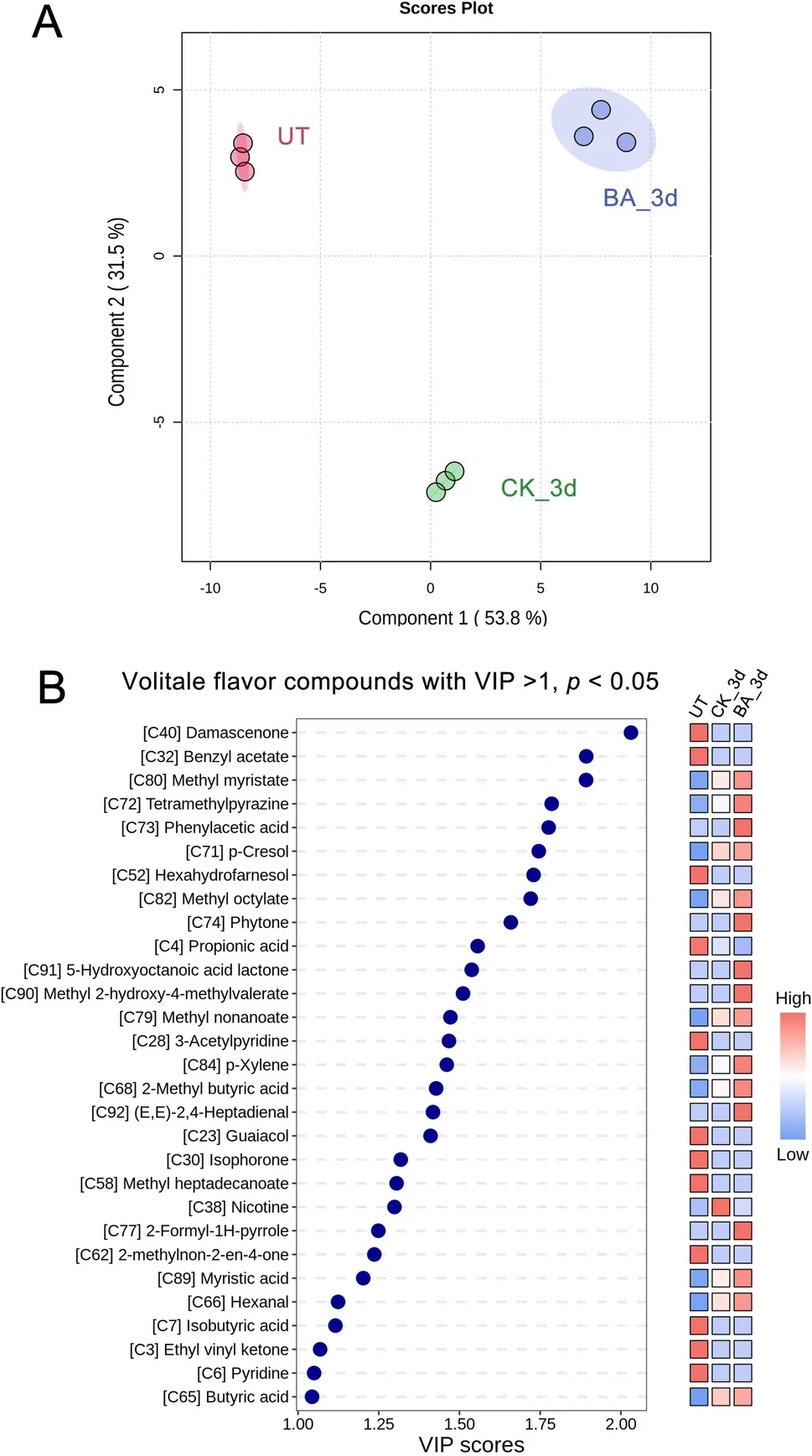
Identification of key differential volatile compounds among the UT, CK_3d, and BA_3d groups. **A** PLS-DA score plot of volatile profiles. **B** Twenty-nine key differential volatile compounds selected based on VIP > 1 and Kruskal-Wallis test with FDR *p* < 0.05. The heatmap shows the relative abundances of individual compounds across the three groups

The PLS-DA model was applied to identify key differential volatile flavor compounds among UT, CK_3d, and BA_3d samples (Fig. 3). The score plot (Component 1 = 53.8%, Component 2 = 31.5%) showed that the three groups were clearly separated with distinguishable flavor characteristics (Fig. 3A). The two component PLS-DA model exhibited strong goodness of fit and predictive ability, with R^2^ = 0.9944 and Q^2^ = 0.9841 (Figure S2A). Model reliability was further assessed using a 1,000-permutation test, which yielded *p* = 0.014 (14/1,000 permutations), suggesting a low risk of overfitting (Figure S2B).

A total of 40 volatile compounds with VIP > 1 were initially identified by PLS-DA. Among them, 29 compounds also showed significant differences among three groups based on Kruskal-Wallis test (FDR *p* < 0.05) and were therefore retained as key differential volatile compounds (Fig. 3B & Supplementary Table S2). These compounds showed distinct treatment-dependent accumulation patterns. In particular, [C72] tetramethylpyrazine, [C73] phenylacetic acid, [C74] phytone, [C77] 2-formyl-1 H-pyrrole, [C80] methyl myristate, [C82] methyl octylate, [C90] methyl 2-hydroxy-4-methylvalerate, [C91] 5-hydroxyoctanoic acid lactone, and [C92] (E, E)-2,4-heptadienal were relatively enriched in the BA group. Tetramethylpyrazine is associated with roasted and nutty notes (Al-Khalili et al. 2025), phenylacetic acid contributes honey-like and sweet notes (Majcher et al. 2014), and phytone is characterized by herbal and floral notes (Zhong et al. 2023). In addition, methyl myristate and methyl octylate are ester compounds that may contribute fruity and floral aroma characteristics (Chen et al. 2026). The enrichment of these compounds was consistent with the improved aroma quality, aroma volume, and sweetness observed in the BA group.

The enrichment of tetramethylpyrazine and phenylacetic acid in the BA group may be related to the coordinated transformation of carbohydrate- and amino acid-derived precursors during *Bacillus* dominated fermentation (Men et al. 2026; Shi et al. 2023). The observed degradation of starch, protein, and cellulose in the BA group may increase the availability of fermentable carbon sources and free amino acid precursors. In this context, carbonyl intermediates derived from carbohydrate metabolism may participate in reactions with amino-containing intermediates derived from amino acid metabolism, thereby favoring the formation of nitrogen-containing heterocyclic compounds such as tetramethylpyrazine (Shi et al. 2023). In parallel, phenylacetic acid may be associated with the transformation of aromatic amino acid precursors, particularly phenylalanine-related intermediates, through putative transamination, decarboxylation, and oxidation steps (Men et al. 2026). Therefore, the accumulation patterns of these compounds provide metabolite-level evidence consistent with the proposed contribution of *Bacillus* associated primary metabolism to aroma formation. In addition, various esters such as [C80] methyl myristate, [C82] methyl octylate, and [C69] γ-butyrolactone were present at noticeably higher levels in the BA group (Fig. 3B), suggesting that BA fermentation possesses stronger ester synthesis and aroma generation capacity. As is well known, ester compounds mostly present fruity and floral odors (Chen et al. 2026), which may help improve the fullness and sweetness of tobacco smoke.

In contrast, [C23] guaiacol, [C4] propionic acid, [C4] propionic acid, [C30] isophorone, and [C71] p-cresol showed relatively high abundances in the CK or UT groups. Notably, propionic acid and isobutyric acid exhibit a pungent sour odor (Faccia et al. 2018), and excessive concentrations may aggravate sour and offensive flavors in smoke. Similarly, excess guaiacol and p-cresol tend to produce an irritating smoked odor (Wang et al. 2018). The accumulation of these components may be potentially associated with the increased irritation and off-flavors in the CK group. Furthermore, [C38] nicotine was markedly enriched in the CK group, which may cause potential negative effects such as enhanced oral and throat irritation as well as weakened sweetness, together with widespread health risks (Arendt-Nielsen et al. 2022). In contrast, the nicotine content in the BA group remained at a level similar to that in the UT group. These results indicate that fermentation inoculated with *B. subtilis* DB-15 shows distinct directionality in regulating tobacco volatile flavor compounds. On the one hand, it induces the enrichment of abundant high-quality flavor components such as esters and heterocycles, potentially contributing to improved aroma quality and volume. On the other hand, it inhibits the accumulation of alkaloids, some short-chain fatty acids, and phenols, thereby effectively reducing smoke irritation and off-flavors. By contrast, water-only treatment may promote the accumulation of potential undesirable flavor components and harmful substances such as nicotine.

### Microbial community succession and characteristic microorganisms identification

#### Analysis of microbial community succession

**Fig. 4:**
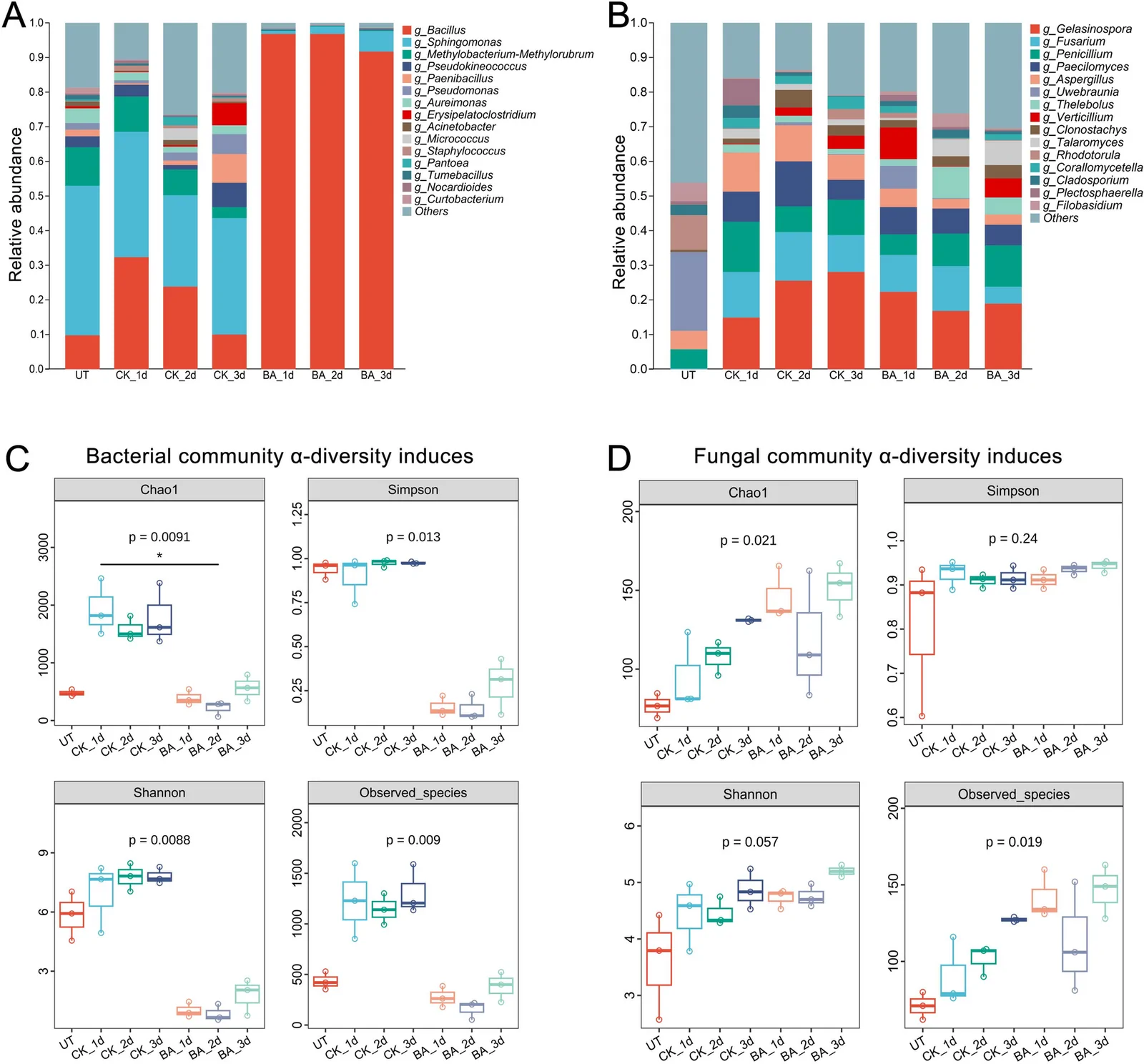
Succession and diversity of microbial communities during fermentation. **A** Bacterial community composition at the genus level. **B** Fungal community composition at the genus level. **C** α-Diversity indices (Chao1, Simpson, Shannon, Observed species) of bacterial communities. **D** α-Diversity indices of fungal communities

The evolution of flavor compounds during fermentation is fundamentally driven by shifts in microbial community structure and metabolic function. Accordingly, the impact of inoculating *B. subtilis* DB-15 on the tobacco microecosystem were further investigated. For the genus level of bacterial community (Fig. 4A), the untreated tobacco leaves (UT group) were dominated by *Sphingomonas*, *Methylobacterium*-*Methylorubrum*, and *Bacillus*. The bacterial community structure of the water-sprayed group changed to some extent but remained largely similar to that of the UT group. Specifically, the relative abundance of *Sphingomonas* decreased slightly but remained dominant, whereas the abundance of *Bacillus* rose sharply on day 1 and then declined continuously to a level comparable with that in the UT group as fermentation proceeded. In addition, water spraying slightly reduced the relative abundances of *Methylobacterium*-*Methylorubrum* and *Aureimonas*, while increasing those of *Pseudokineococcus*, *Paenibacillus*, and *Erysipelatoclostridium* (Fig. 4A). In contrast, the inoculated *Bacillus* in the BA group rapidly became the absolutely dominant genus during fermentation, with its relative abundance stably maintained at above 90%. This result indicated that *B. subtilis* DB-15 successfully established competitive dominance, reshaped the bacterial community structure, and markedly inhibited the growth of other bacterial genera. Furthermore, the relative abundance of *Sphingomonas* in the BA group increased continuously with fermentation.

The fungal community structure showed a different pattern of variation (Fig. 4B). The UT group was dominated by *Uwebraunia* and *Filobasidium*, whereas the fungal community structures of the CK and BA groups were closely similar and both clearly differed from that of the UT group. This finding suggested that water addition may be the primary factor driving fungal community succession, while inoculation with *B. subtilis* DB-15 exerted a relatively limited effect on fungal communities. Both the CK and BA groups showed clearly enrichment of genera including *Gelasinospora*, *Fusarium*, *Penicillium*, *Paecilomyces*, and *Aspergillus* (Fig. 4B). These fungi are commonly reported as ubiquitous taxa in tobacco cultivation and processing environments, and their increased abundance may be related to the improved growth conditions and enhanced proliferation of indigenous fungi caused by water addition. Notably, although these fungi may participate in partial macromolecule degradation during fermentation (Yélamos et al. 2025), the lack of effective targeted regulation easily leads to the production of undesirable flavors and quality deterioration, which may be potentially responsible for the aggravated off-flavors in the CK group.

#### Microbial community diversity and identification of characteristic microorganisms

Microbial community diversity directly reflects the stability and functional potential of the fermentation system (Nikoloudaki et al. 2024). The α-diversity indices of microbial communities were further analyzed to evaluate the effects of different treatments on community richness and evenness. For the bacterial community (Fig. 4C-D), the Shannon, Simpson, and Observed species indices in the BA group were significantly lower than those in the UT group during fermentation (*p* < 0.05) (Fig. 4C), whereas water spraying increased the Chao1, Shannon, and Observed species indices of the bacterial community. These results indicated that inoculation with *B. subtilis* DB-15 significantly reduced the richness and diversity of the tobacco bacterial community, forming a low-diversity community structure dominated by the inoculated strain. In contrast, water spraying enhanced community diversity and richness. For the fungal community (Fig. 4D), the Chao1 and Observed species indices in the CK and BA groups were significantly higher than those in the UT group (*p* < 0.05), and the Shannon index was also noticeably higher than that in the UT group (*p* = 0.057), while no significant differences were observed between the CK and BA groups. This further demonstrated that water addition was the main factor driving the improvement of fungal community richness and diversity, whereas inoculation with *B. subtilis* DB-15 exerted a relatively limited effect on fungal α-diversity. Collectively, the significant reduction in bacterial community diversity and the non-specific elevation of fungal community diversity following BA treatment further confirmed that *B. subtilis* DB-15 fermentation achieves its functional advantages mainly by reshaping the bacterial community structure, with a weak impact on fungal communities.

**Fig. 5:**
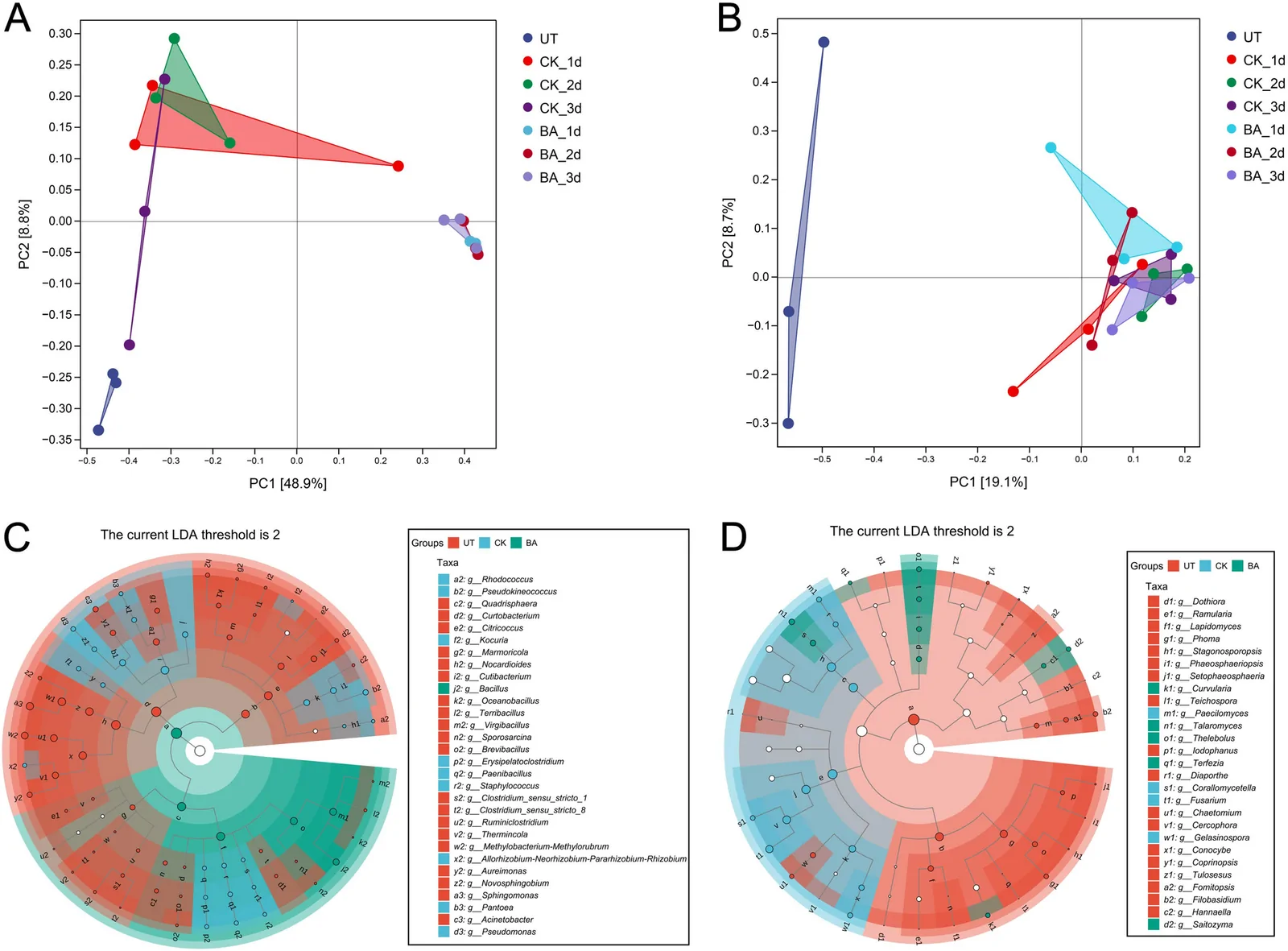
β-diversity and characteristic microorganisms identified by LEfSe analysis. **A** Bacterial PCoA plot. **B** Fungal PCoA plot. **C** LEfSe analysis of bacterial communities (LDA score > 2). **D** LEfSe analysis of fungal communities (LDA score > 2)

To further explore the degree of community differentiation and identify characteristic taxa in different treatment groups, PCoA and LEfSe analysis were conducted (Fig. 5). PCoA-based β-diversity analysis showed distinct differentiation patterns for bacterial and fungal communities. In the bacterial community (Fig. 5A), the UT and BA groups were obviously separated mainly along the PC1 axis (48.9%), while the UT and CK groups were separated mainly along the PC2 axis (only 8.8%). These results indicated that the community structure of the CK group was overall more similar to that of the UT group, whereas samples in the BA group were tightly clustered along the PC1 axis to form an independent cluster, suggesting that inoculation with *B. subtilis* DB-15 was the key factor driving the fundamental and directional alteration of the bacterial community. For the fungal community (Fig. 5B), the UT group was clearly separated from the CK and BA groups, but samples from the CK and BA groups were highly clustered, further verifying that water addition was the dominant factor affecting fungal community succession, and BA inoculation caused no significant additional disturbance.

Finally, LEfSe analysis (LDA > 2) was conducted to further identify the characteristic microorganisms in each treatment group (Fig. 5C-D). The characteristic bacterium in the BA group was *Bacillus*, which was fully consistent with the dominant position of the inoculated strain (Fig. 5C). The characteristic bacteria in the CK group included *Pseudokineococcus* and *Corynebacterium*, while the UT group was enriched with environmental bacteria such as *Sphingomonas* and *Acinetobacter*. For characteristic fungi (Fig. 5C), the features in the CK and BA groups were mainly common processing-environment fungi such as *Fusarium* and *Penicillium* (Fig. 5D), whereas the UT group was characterized by taxa commonly found in low-moisture tobacco leaves, such as *Dothiora* and *Ramularia*.

### Prediction of microbial community functional potential based on PICRUSt2

**Fig. 6:**
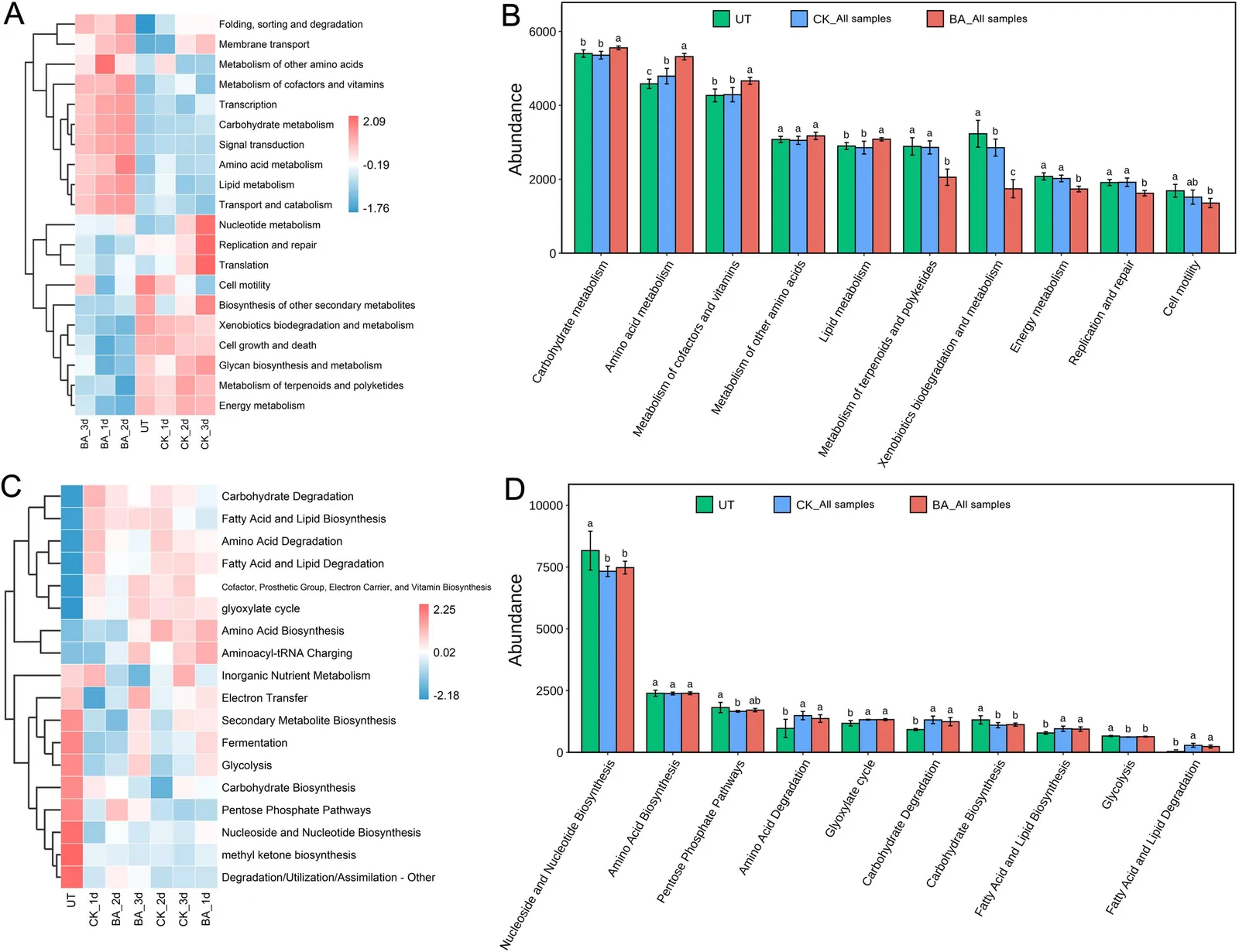
Microbial metabolic function prediction during fermentation. **A** Heatmap showing the top 20 predicted bacterial KEGG level-2 pathways. **B** Duncan’s multiple comparison of the top 10 predicted bacterial metabolic pathways. **C** Heatmap showing the top 20 predicted fungal MetaCyc pathways. **D** Duncan’s multiple comparison of the top 10 predicted fungal metabolic pathways. Note: different lowercase letters (a, b, c) indicate significant differences among groups at the same time point (*p* < 0.05, one-way ANOVA followed by Duncan’s multiple comparison test)

Differences in community structure are often accompanied by variation in microbial functional potential. To explore the intrinsic mechanism by which microorganisms drive the transformation of flavor compounds, the metabolic potentials of bacterial and fungal communities were predicted using PICRUSt2 (Xu et al. 2026). Heatmaps illustrated the dynamic abundance patterns of the top 20 KEGG level-2 functions during fermentation, and Duncan multiple comparison was performed for the top 10 metabolic functions among the three groups (Fig. 6).

For bacterial functional potential, the predicted relative abundances of pathways assigned to carbohydrate metabolism and amino acid metabolism were higher in the BA group than in the UT and CK groups throughout fermentation (Fig. 6A,B). This functional pattern was consistent with the marked degradation of starch, protein, and cellulose observed in the BA group, as well as with the enrichment of aroma-related esters, alcohols, and nitrogen-containing heterocyclic compounds (Ma et al. 2026; Shu et al. 2026). A plausible explanation is that carbohydrate degradation increases the availability of fermentable carbon sources and carbonyl intermediates, whereas amino acid metabolism supplies amino-containing and aromatic precursors. The coupling of these primary metabolic processes may provide favorable precursor conditions for the formation of aroma-active compounds. For example, the formation of tetramethylpyrazine may be favored by the interaction between carbonyl intermediates and amino-containing precursors, while the accumulation of phenylacetic acid may be associated with the conversion of phenylalanine-related intermediates (Zhou et al. 2024). Therefore, the higher predicted abundances of carbohydrate- and amino acid-metabolism pathways in the BA group provide a functional ecological explanation for the observed enrichment of tetramethylpyrazine, phenylacetic acid, and other favorable aroma compounds during BA fermentation (Bleicher et al. 2022). Notably, the decreased predicted abundance of terpenoid and polyketide metabolism and other secondary metabolic pathways in the BA group suggests that the flavor improvement of BA fermentation may be more closely associated with shifts in primary metabolism rather than secondary metabolism (Fig. 6A-B). In contrast, the CK and UT groups showed no obvious advantage in the predicted bacterial functional pathways. This pattern may partly account for the relatively simpler flavor profile and inferior sensory performance of the CK group.

The successional pattern of fungal metabolic potential was distinctly different from that of bacteria (Fig. 6C,D). The heatmap of the top 20 MetaCyc pathways showed that the abundance of nucleoside and nucleotide biosynthesis in the UT group was obviously higher than in the CK and BA groups (Fig. 6C), whereas the abundances of carbohydrate degradation, amino acid degradation, and fatty acid and lipid degradation in the CK and BA groups were obviously higher than in the UT group (Fig. 6C), with highly similar predicted levels between CK and BA. Duncan’s multiple comparison further verified that the abundance of nucleoside and nucleotide biosynthesis in the UT group was significantly higher than in the CK and BA groups (*p* < 0.05), while no significant differences were observed between CK and BA in the upregulated degradation pathways (Fig. 6D). These results suggest that water addition was the dominant factor associated with shifts in fungal functional potential, whereas *B. subtilis* DB-15 inoculation exerted relatively limited additional effects on the predicted fungal pathways. Combined with the above flavor results, the elevated predicted fungal pathways related to macromolecule degradation in the CK group may promote the accumulation of potential undesirable flavors such as propionic acid and p-cresol, thereby intensifying sourness, off-flavors, and irritation in smoke. In the BA group, bacteria-dominated metabolic pathways predominate, and fungi act only as background microbiota with minimal negative impacts on overall flavor quality, which may partially explain the significantly better flavor coordination in the BA group than in the CK group.

Taken together, the present results support a putative mechanism in which inoculated B. subtilis DB-15 establishes bacterial dominance and promotes a community functional profile enriched in carbohydrate- and amino acid-metabolism categories. The extracellular enzyme activities of DB-15 and the reduced contents of starch, protein, and cellulose suggest enhanced release and transformation of carbon- and nitrogen-containing precursors in tobacco leaves. These precursor changes may facilitate the generation of favorable volatile compounds, including esters, aromatic compounds, and nitrogen-containing heterocycles, thereby contributing to improved aroma quality, aroma volume, sweetness, and sensory coordination. The markedly different fungal profiles between UT and water-treated samples, together with the similar fungal functional profiles in CK and BA, further suggest that the principal mechanism of BA fermentation is more closely linked to the Bacillus-dominated bacterial community than to targeted remodeling of fungal metabolism. It should be noted that PICRUSt2 analysis provides an inference of microbial functional potential from 16 S rRNA gene and ITS amplicon data. Therefore, the predicted pathway shifts should be interpreted together with the observed macromolecule degradation and volatile-compound profiles, rather than as direct evidence of in situ gene expression or metabolic activity. Future metagenomic, metatranscriptomic, targeted enzyme-activity, or isotope-tracing analyses will help further validate the proposed functional roles of the microbial community during tobacco fermentation.

### Correlation analysis of sensory quality, key flavor compounds, and characteristic microorganisms

**Fig. 7:**
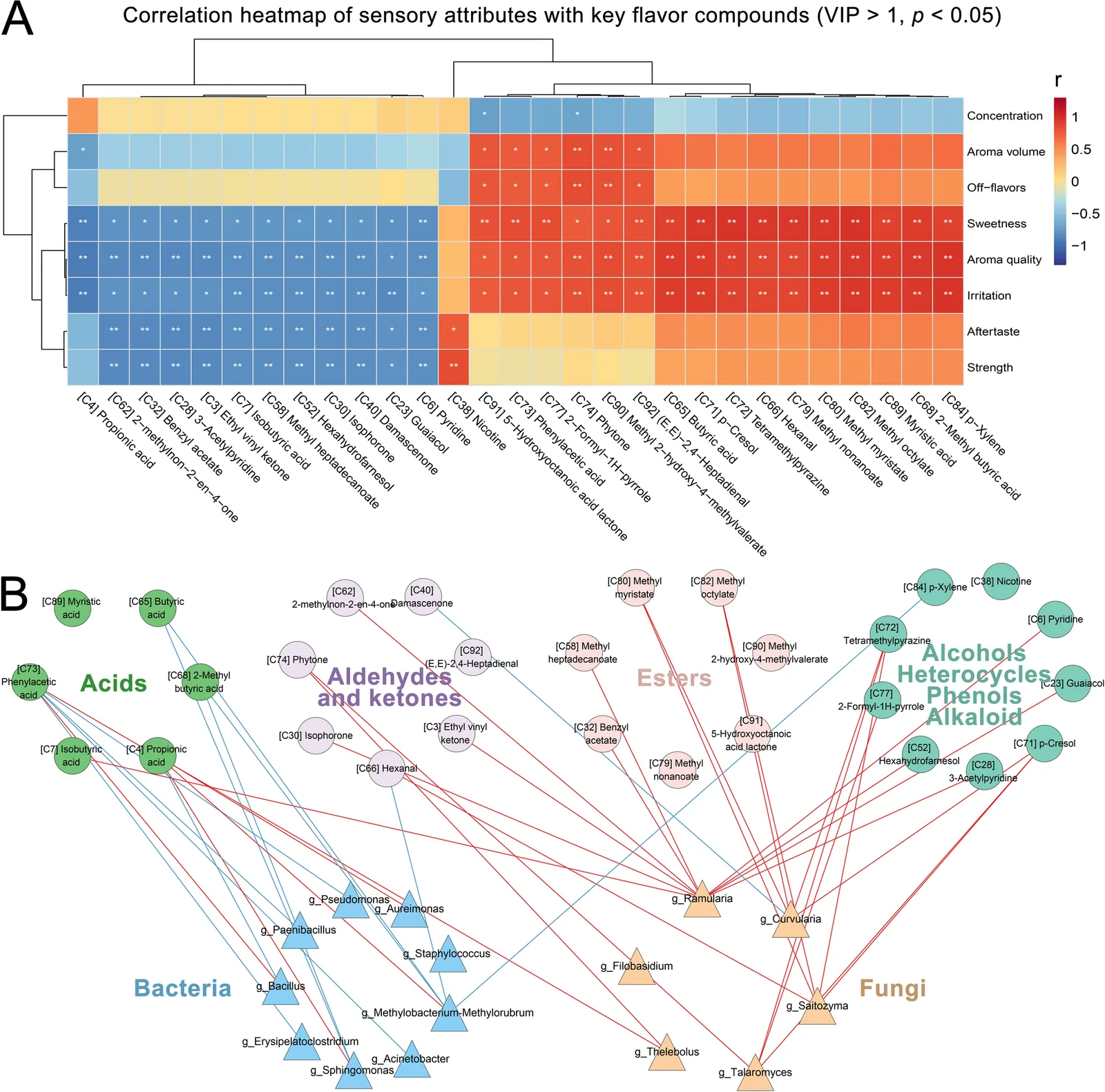
Correlation analysis of sensory attributes, key volatile compounds, and characteristic microorganisms. **A** Correlation heatmap between sensory attributes and 29 key volatile compounds (VIP > 1, *p* < 0.05) based on Spearman correlation coefficients. Red and blue colors indicate positive and negative correlations, respectively. Asterisks within the heatmap cells indicate statistically significant correlations (*p < 0.05, **p < 0.01). **B** Bipartite network visualizing the significant correlations between key volatile compounds (VIP > 1, *p* < 0.05) and characteristic microorganisms identified by LEfSe (LDA score > 2). Edges represent significant Spearman correlations with |r| > 0.8 and FDR *p* < 0.05. Red and blue edges indicate positive and negative correlations, respectively

To explore the intrinsic relationships between sensory quality and volatile composition, a correlation heatmap was generated between sensory attributes and the 29 key differential volatile compounds identified at the fermentation endpoint (VIP > 1 and Kruskal-Wallis test *p* < 0.05) (Fig. 7A). From the correlation between sensory attributes and flavor substances, various esters including [C79] methyl nonanoate, [C80] methyl myristate, [C82] methyl octylate, [C90] methyl 2-hydroxy-4-methylvalerate, and [C91] 5-hydroxyoctanoic acid lactone were significantly positively correlated with aroma volume, aroma quality, off-flavors, and irritation (Fig. 7A). Esters mostly present fruity, sweet, floral, and mild aromatic notes (Holt et al. 2019), which can improve the aromatic richness and smoothness of smoke, may help mask off-flavors, reduce irritation, and enhance aroma quality and volume, and may serve as important aroma contributors for improving tobacco sensory quality. In addition, [C73] phenylacetic acid, [C74] phytone, [C77] 2-formyl-1 H-pyrrole, [C90] methyl 2-hydroxy-4-methylvalerate, [C91] 5-hydroxyoctanoic acid lactone, [C92] (E, E)-2,4-heptadienal, and [C72] tetramethylpyrazine also showed significant positive correlations with sweetness, off-flavors, irritation, aroma volume, and aroma quality (Fig. 7A), suggesting that these compounds may also contribute to improved aroma fullness, enhanced aroma quality, reduced off-flavors, and decreased irritation. Furthermore, [C38] nicotine was significantly positively correlated with strength, while [C7] isobutyric acid and [C4] propionic acid were significantly negatively correlated with irritation (a higher irritation score indicates weaker sensory irritation), suggesting they are key contributors to increased smoke irritation. Previous studies have shown nicotine determines tobacco strength (McKinney et al. 2014), and these two acids with pungent sour odors may enhance smoke irritation (Faccia et al. 2018). The nicotine content in the CK group was higher than that in the UT and BA groups, and may be a key factor leading to higher smoke strength, stronger irritation, and more obvious off-flavors under insufficient buffering from sweet and fruity compounds. In addition, [C3] ethyl vinyl ketone, [C6] pyridine, [C7] isobutyric acid, [C23] guaiacol, [C30] isophorone, and other compounds were significantly negatively correlated with irritation, aroma volume, and aroma quality (Fig. 7A). Their relative enrichment in the UT and CK groups may be potential factors aggravating offensive odors and irritation in smoke.

To further explore the associations between characteristic microorganisms (LEfSe, LDA > 2) and key volatile compounds (VIP > 1), a bipartite network was generated using Spearman’s correlation with |r| > 0.8 and FDR *p* < 0.05 (Fig. 7B). It is worth noting that acids were more closely associated with bacteria, while alcohols, heterocycles, and phenols were more closely related to fungi (Fig. 7B). Among them, the inoculated *Bacillus* was significantly positively correlated with [C73] phenylacetic acid (honey-sweet aroma) (Majcher et al. 2014) and significantly negatively correlated with [C4] propionic acid and [C50] 4-hydroxy-β-damascone. These results suggest that the inoculated Bacillus may contribute to improved sweetness, aroma volume, and aroma quality, as well as reduced off-flavors and irritation, through its positive association with sweet aromatic compounds such as phenylacetic acid and negative association with potential undesirable compounds such as propionic acid. In addition, indigenous bacteria including *Sphingomonas* and *Methylobacterium*-*Methylorubrum* were significantly negatively correlated with [C65] butyric acid, [C68] 2-methyl butyric acid, and [C70] phenylacetaldehyde, and barely promoted the formation of high-quality flavor compounds (Fig. 7B). *Ramularia* was significantly positively correlated with [C3] ethyl vinyl ketone, [C6] pyridine, [C7] isobutyric acid, and [C23] guaiacol (Fig. 7B), which were negatively correlated with multiple positive sensory indicators, suggesting that the proliferation of this fungus in the UT and CK groups may be unfavorable to sensory quality optimization. Overall, the correlation analysis identified Bacillus-centered associations as an important characteristic of BA fermentation. Together with the compositional and sensory results, these findings support a potential link between Bacillus dominance, the enrichment of favorable volatile compounds, and improved sensory coordination.

## Conclusion

This study confirmed that inoculation with *B. subtilis* DB-15 could effectively degrade macromolecules and directionally regulate volatile flavor metabolism in tobacco leaves, thereby significantly improving sensory quality by increasing pleasant aromas and reducing irritation and off‑flavors. Microbiomics analysis revealed that the inoculated strain reshaped the bacterial community structure and became the dominant genus, while fungal community succession was mainly affected by water addition. Functional prediction and correlation network analysis jointly indicated that *B. subtilis* DB-15 fermentation was associated with enhanced primary metabolic potential and the accumulation of favorable flavor compounds such as tetramethylpyrazine and phenylacetic acid, together with reduced levels of several irritant components. These findings illustrate the coordinated regulation mechanism of microbiota and flavor metabolism during fermentation, and provide a feasible green approach for upgrading low‑quality tobacco. As PICRUSt2-based functional analysis reflects predicted community functional potential rather than direct pathway activity, future studies integrating metagenomics, metatranscriptomics, targeted enzyme assays, and metabolomics will further validate the key metabolic pathways and optimize fermentation parameters for industrial application.

## Supplementary Information

Below is the link to the electronic supplementary material.


Supplementary Material 1.



Supplementary Material 2.


## Data Availability

The datasets presented in this study can be available in the article and its Supplementary materials, and further inquiries can be directed to the corresponding author or the first author. The raw sequencing reads of microbial amplicon have been deposited in the China National Center for Bioinformation (CNCB) database under BioProject ID PRJCA068606.
