# Supplementary material for: Insights into volatile flavoromics and microbiomics for sensory quality improvement of flue-cured tobacco leaves fermented with Bacillus subtilis DB-15
Source: Bioresour Bioprocess. 2026 Aug 31;13(1):125. doi: 10.1186/s40643-026-01124-2 (PMC13530059; doi:10.1186/s40643-026-01124-2)
Supplement: Supplementary file 1 [file 40643_2026_1124_MOESM1_ESM.docx]

Insights into volatile flavoromics and microbiomics for sensory quality improvement of flue-cured tobacco leaves fermented with *Bacillus subtilis* **DB**-15

**——Supplementary Materials**

Yanxu Guo ^a^, Junxi Wang ^a^, Guiyuan Huang ^a^, Ningning Hou ^a^, Haiyang Pan ^a^, Wei Ding ^a,*^

^a^ Technical Research Center, China Tobacco Sichuan Industrial Co., Ltd., 56 Chenglong Road, Chengdu, 610000, China

**^*^Corresponding author**: Wei Ding

**E-mail**: dingwei198106@163.com

**Affiliation**: Technical Research Center, China Tobacco Sichuan Industrial Co., Ltd., 56 Chenglong Road, Chengdu, 610000, China

**Figure S1 Chromatogram and retention time of C7-C30 n-alkane mixtures.**

**Figure S2 The R2, Q2 and 1000-fold permutation tests p values of PLS-DA**
